# Examining the immunoepigenetic-gut microbiome axis in the context of self-esteem among Native Hawaiians and other Pacific Islanders

**DOI:** 10.3389/fgene.2023.1125217

**Published:** 2023-04-19

**Authors:** Celyna Y. Becerra, Riley K. Wells, Braden P. Kunihiro, Rosa H. Lee, Lesley Umeda, Nina P. Allan, Noelle C. Rubas, Trevor A. McCracken, Chandler K. L. Nunokawa, Ming-Hao Lee, Felix Gerard S. Pidlaoan, Krit Phankitnirondorn, Christian K. Dye, Brennan Y Yamamoto, Rafael Peres, Ruben Juarez, Alika K. Maunakea

**Affiliations:** ^1^ Department of Anatomy, Biochemistry, and Physiology, John A. Burns School of Medicine, Honolulu, HI, United States; ^2^ IDeA Networks of Biomedical Research Excellence (INBRE), University of Hawaii at Manoa, Honolulu, HI, United States; ^3^ Department of Molecular Biosciences and Bioengineering, University of Hawaii at Manoa, Honolulu, HI, United States; ^4^ Department of Environmental Health Sciences, Columbia University Irving Medical Center, NY, NY, United States; ^5^ Department of Economics, University of Hawaii at Manoa, Honolulu, HI, United States; ^6^ University of Hawaii Economic Research Organization (UHERO), University of Hawaii at Manoa, Honolulu, HI, United States

**Keywords:** health disparities, depressive symptoms, epigenetic aging, self-esteem, immune system, gut microbiome, DNA methylation, Native Hawaiians and other Pacific Islanders

## Abstract

**Introduction:** Native Hawaiian and other Pacific Islander (NHPI) populations experience higher rates of immunometabolic diseases compared to other racial-ethnic groups in Hawaii. As annual NHPI mortality rates for suicide and type 2 diabetes mellitus (T2DM) exceed those of the state as a whole, understanding the social and biological mechanisms underlying these disparities are urgently needed to enable preventive strategies.

**Methods:** A community-based approach was used to investigate the immunoepigenetic-gut microbiome axis in an NHPI-enriched cohort of Oahu residents (*N* = 68). Self-esteem (SE) data was collected using a modified Rosenberg self-esteem (SE) assessment as a proxy measure for mental wellbeing in consideration for cultural competency. T2DM status was evaluated using point-of-care A1c (%) tests. Stool samples were collected for 16s-based metagenomic sequencing analyses. Plasma from blood samples were isolated by density-gradient centrifugation. Peripheral blood mononuclear cells (PBMCs) were collected from the same samples and enriched for monocytes using negative selection techniques. Flow-cytometry was used for immunoprofiling assays. Monocyte DNA was extracted for Illumina EPIC array-based methylation analysis.

**Results:** Compared to individuals with normal SE (NSE), those with low SE (LSE) exhibited significantly higher plasma concentrations (pg/ml) of proinflammatory cytokines IL-8 (*p* = 0.051) and TNF-α (*p* = 0.011). Metagenomic analysis revealed that the relative abundance (%) of specific gut bacteria significantly differed between SE groups - some of which directly correlated with SE scores. Gene ontology analysis revealed that 104 significantly differentially methylated loci (DML) between SE groups were preferentially located at genes involved in immunometabolic processes. Horvath clock analyses indicated epigenetic age (Epi-Age) deceleration in individuals with LSE and acceleration in individuals with NSE (*p* = 0.042), yet was not reproduced by other clocks.

**Discussion:** These data reveal novel differences in the immunoepigenetic-gut microbiome axis with respect to SE, warranting further investigation into its relationship to brain activity and mental health in NHPI. Unexpected results from Epi-Age analyses warrant further investigation into the relationship between biological age and disparate health outcomes among the NHPI population. The modifiable component of epigenetic processes and the gut microbiome makes this axis an attractive target for potential therapeutics, biomarker discovery, and novel prevention strategies.

## Introduction

Native Hawaiians and other Pacific Islanders (NHPI) are burdened by a disproportionately high prevalence of and mortality to chronic, preventable diseases including those linked with immunometabolic dysregulation such as diabetes and obesity (Kaholokula et al., 2010; Nakagawa et al., 2015; Ing et al., 2019; Uchima et al., 2019). These health disparities extend to mental health and have been exacerbated by the COVID-19 pandemic (Juarez et al., 2022). In addition, clinically underserved NHPI communities are reported to be three times less likely to receive mental health services and treatment compared to Non-Hispanic White People (U.S Department of Health and Human Services, Office of Minority Health, 2019). Furthermore, NHPI have a higher prevalence of having depressed mood, suicidal thoughts and attempts, and the usage of illicit drugs (Yuen et al., 2000; Subica and Wu, 2018). While existing literature seeks to characterize the biological basis of mood disorders, few have extended further research into mental health disparities of the NHPI population, including assessments of self-esteem (SE).

SE is an important cognitive component of mental health applied in several studies. The Rosenberg Self-Esteem Scale (SE score) is the most widely used measure of global SE with evidence of generalizability across ethnic groups (Rosenberg, 1962; Miyamoto et al., 2001). It is a 10-item scale consisting of 5 positively- and 5 negatively-phrased statements. Participants respond with a “strongly disagree,” “disagree,” “agree,” or “strongly agree,” with a score of 0, 1, 2, and 3, respectively, for each statement on the Likert scale, with the total score ranging from 0 to 30.

While SE is not clinically defined by the Diagnostic and Statistical Manual (DSM-5; Uher et al., 2014), its relationship with health outcomes has been used extensively in biomedical research. Existing literature implicates low SE (LSE) as an indicator for various depressive disorders (Steiger et al., 2014; Park and Yang, 2017) and anxiety disorders (Rosenberg, 1962; Peñate et al., 2020). While conventionally perceived as a diagnostic tool for mental illness, SE functions as an independent factor of overall wellbeing (Centers for Disease Control and Prevention, 2018; Niveau et al., 2021), extending to its increasingly recognized association with physical health outcomes (Trzesniewski et al., 2006). LSE is reportedly associated with chronic illnesses such as metabolic (Luyckx et al., 2016) and cardiovascular disorders (O’Donnell et al., 2008), which are thought to stem from inflammatory dysregulation (Liberale et al., 2021). Such studies have prompted further investigation of the gut microbiome, as it modulates systemic inflammation, and as evidence continues to grow around its association with mental health.

Indeed, dysbiosis of the gut microbiome has been associated with depression (Jiang et al., 2015; Madison and Kiecolt-Glaser, 2019; Johnson, 2020). Fecal transplantation from a healthy to a depressed individual has demonstrated sufficiency in reducing major depressive symptoms (Doll et al., 2022). Preclinical studies have shown that major depressive symptoms can be induced in mice *via* fecal transplantation (Kelly et al., 2016; Zheng et al., 2016). Expanding research in this study area approaches a consensus that changes to the gut microbiome result in far-reaching changes to brain chemistry and activity (Foster and McVey Neufeld, 2013). At the level of host physiology, these changes are thought to be partially mediated by inflammatory pathways.

Compromised immune function and increased serum levels of proinflammatory cytokines have been found in depressed individuals compared to unaffected controls (Howren et al., 2009; Schmidt et al., 2014; Lee and Giuliani, 2019). Furthermore, epigenetic processes have been observed to influence depressive symptoms (Nemoda et al., 2015; Emeny et al., 2018; Zhu et al., 2019). While previous studies have described epigenetic regulation of genes commonly linked with depressive symptoms (Carlberg et al., 2014; Booij et al., 2015; Murgatroyd et al., 2015; Maud et al., 2018), little is known about the relationships between epigenetic regulation of immune cell function and inflammation in the context of gut microbial dysbiosis and SE.

The immunoepigenetic-gut microbiome axis involves interactions between the gut microbiome, immune function, and inflammation that underlies immunometabolic dysregulation (Martin et al., 2018). Maintenance of microbial health, defined by abundant diversity and enrichment of microbial short-chain fatty acid (SCFA) producers as examples, associates with optimal immune function. This favorable microbial composition fortifies the colonic epithelial lining and, in turn, buffers intestinal inflammation through various microbial-induced protective mechanisms. However, in a state of microbial dysbiosis, the intestinal barrier becomes susceptible to opportunistic microbial inhabitants. Dysbiosis subsequently deteriorates the epithelium, propagates localized inflammation, and provides inflammatory microbial byproducts with circulatory access. The consequence of prolonged dysbiosis is the reprogramming of systemic immune cells to proinflammatory states that ultimately establish and maintain chronic inflammation, which contributes to the progression of various non-communicable diseases (Rubas and Maunakea, 2021). The complicated relationship between the gut microbiome and host immunomodulatory physiology may be leveraged in developing therapeutic strategies to address the cognitive facets of mood-related disorders. In this study, we sought to understand the relationships between the gut microbiome, epigenetic regulation of inflammation, and SE, which may explain mental health disparities in the NHPI population.

## Methods

### Collection of biometric and SE data

To quantify self-esteem (SE) within our cohort (*N* = 68), we administered a Rosenberg SE Scale assessment modified in consideration for cultural competency. Cumulative SE scores ranging from 0 to 15 qualified as low SE (LSE) and those ranging from 16 to 30 qualified as normal SE (NSE). Sample questions and corresponding point-value assignments for each response for SE score data collection are provided in Supplementary Table S1. We chose to implement a Rosenberg-adjacent assessment for its reliability across ethnic groups in adult samples and to remain consistent with existing data collected from NHPI-enriched cohorts (Singelis et al., 1999; Robins et al., 2001). The following health measurements were then collected: height, weight, blood pressure, heart rate, and hemoglobin A1c (%; A1CNow+, PTS Diagnostics, IN-USA). Blood was collected from participants (up to 20 mL) at our community sites by venipuncture upon biometric data collection. Patients receiving medical treatment for either type 1 or type 2 diabetes were not included in the study cohort.

### Blood sample processing

Plasma and peripheral blood mononuclear cells (PBMCs) were stratified within 24 h of sample collection using density-gradient centrifugation in SepMate tubes (Stemcell technologies, Canada). Five plasma aliquots (1 mL) from each sample were stored at −80°C until further application. PBMCs were stored at −150°C until the moment of assay performance.

### Quantification of immunometabolic biomarkers

Plasma concentrations of IFN-γ, IL-1β, IL-6, IL-8 (CXCL8), IL-10, MCP-1 (CCL2), TNF-α, and VEGF-A were assessed using the 9-Plex Human ProcartaPlex Panel (ThermoFisher part no. PPX-09, assay MXEPTU6) in accordance with manufacturer provided protocols. Samples were centrifuged at 13,000 × g for 2 min to pellet aggregates. A standard curve was generated using antigen standards provided by the manufacturer. Bead counts below 35 were excluded from analysis. Fluorescent signals were analyzed using the Luminex 200™ instrument (R&D Systems, Inc., Minneapolis, MN, United States). Bio-Plex Manager™ software (Bio-Rad Laboratories, Inc., Hercules, CA, United States) was used for data processing. Glucose levels were measured using Glucose Colorimetric Detection Kit (Thermo Fisher Scientific, Inc., Waltham, MA, United States) upon 15-fold sample dilution. Cortisol was measured using Cortisol Competitive Human ELISA Kit (EIAHCOR), (Thermo Fisher Scientific Inc., Frederick, MD, United States). Adiponectin was measured using Adiponectin Human ELISA Kit (KHP0041), (Thermo Fisher Scientific Inc., Vienna, Austria). Leptin was measured using Leptin Human Instant ELISA™ Kit (BMS2039INST), (Thermo Fisher Scientific Inc., Vienna, Austria), and PYY levels were measured using Human PYY ELISA Kit (EH387RB), (Thermo Fisher Scientific Inc., Carlsbad, CA, United States). All assays were performed using protocols provided by respective manufacturers and was read on a SpectraMax ABS/ABS Plus Microplate Reader (Molecular Devices, San Jose, CA, United States).

### Stool sample collection and processing

Home stool sample collection kits were distributed to each participant. These kits included one sample tube containing RNAlater (5 mL; Thermofisher Scientific, Waltham, MA) as a sample preservative. Instructions for proper sample collection and storage were provided verbally and in print. Samples were submitted by mail or collected by a community research facilitator. Upon receipt, samples were stored at −20°C before nucleic acid purification.

**FIGURE 1:**
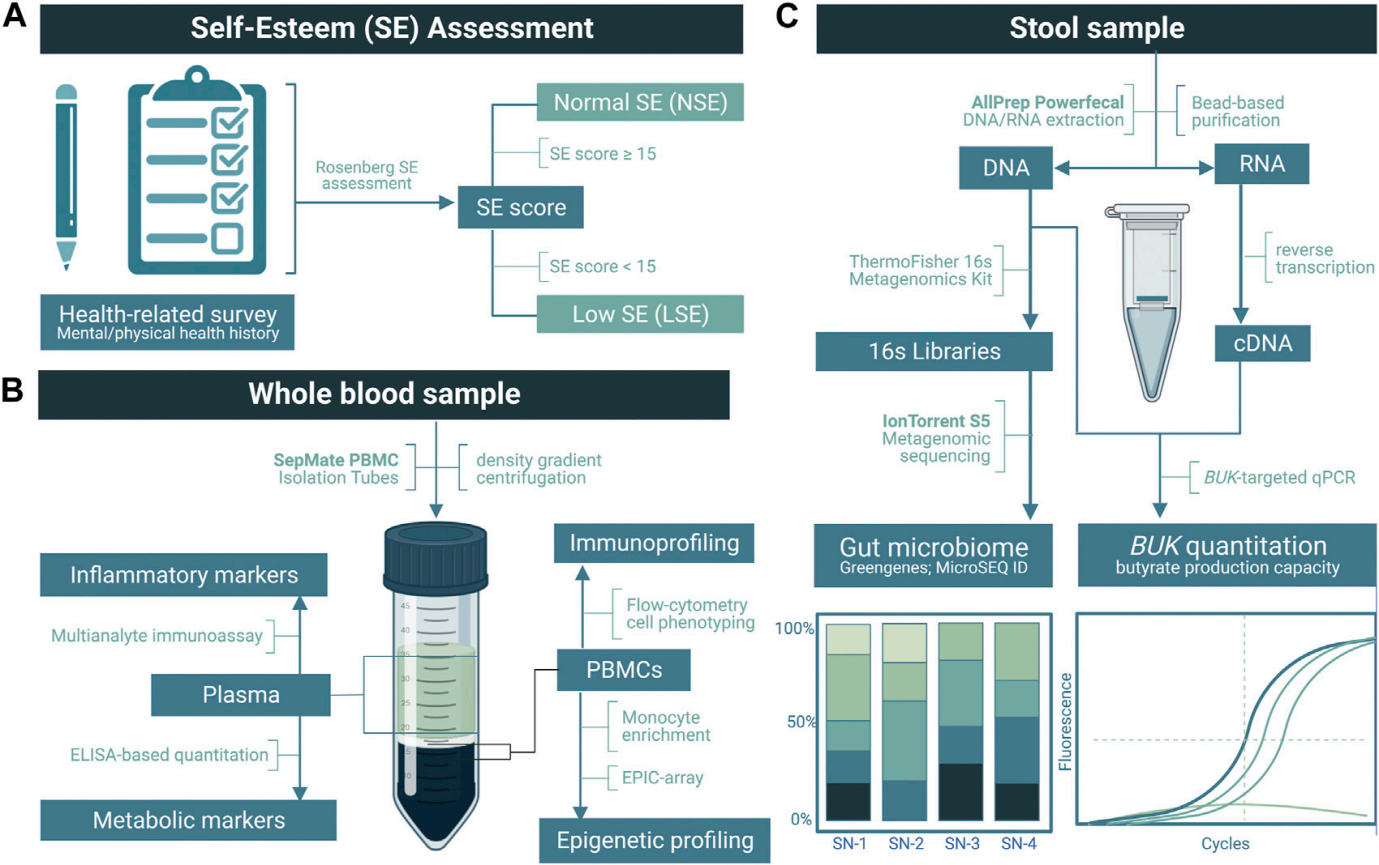
Overview of the methodology used in this study. **(A)** Application of a modified Rosenberg self-esteem (SE) assessment to categorize low SE (LSE) and normal SE (NSE) groups. **(B)** Upon the separation of the plasma, inflammatory and metabolic markers were quantified by multianalyte immunoassay and enzyme-linked immunosorbent assay (ELISA), respectively. From the peripheral blood mononuclear cells (PBMCs), flow cytometry was performed for immunoprofiling, followed by monocyte enrichment and an EPIC array for epigenetic profiling. **(C)** Following nucleic acid purification from stool samples, DNA was used to quantify the relative abundance of gut bacteria *via* metagenomic sequencing and butyrate-kinase (BUK) gene copies *via* real-time PCR (rtPCR). RNA was reverse-transcribed to cDNA for BUK transcript rtPCR.

Stool samples were processed *via* MagMAX Microbiome Ultra Nucleic Acid Isolation Kits (Thermo Fisher Scientific, Inc., Waltham, MA, United States) for simultaneous DNA and RNA extraction, using KingFisher Duo Prime automated extraction system. From each sample, 1–2 μL of purified DNA and RNA was analyzed using the Thermo Fisher NanoDrop Microvolume Spectrophotometer to assess sample quality and the Qubit Fluorometer for quantity (Thermo Fisher Scientific, Inc., Waltham, MA, United States). An overview of workflow methodology for sample processing and subsequent analyses are illustrated in Figure 1.

### 16s metagenomic sequencing

DNA (40 ng) isolated from each stool sample was subjected to polymerase chain reaction (PCR) amplification targeting 16s rDNA hypervariable regions V2-4 and V6-9 as we previously performed (Wells et al., 2022). Briefly, amplicon products were pooled (20 μL per primer set), purified (Agencourt Ampure XP Kit; Beckman Coulter, Brea, CA, United States), and quantified using the Qubit dsDNA BR Assay (ThermoFisher Scientific, Warrington, England). 16s rDNA libraries were prepared from 150 ng of pooled amplicons (Ion Plus Fragment Library Kit; Thermo Fisher Scientific, Austin, TX, United States) and barcoded using Ion Xpress Barcode Adapters (Life Technologies, Carlsbad, CA, United States). DNA libraries were pooled (80 pmol from up to 60 libraries) and loaded onto Ion 530™ chips (Ion S5 Next-Generation Sequencing System) in preparation for sequencing.

16s Metagenomics Kit analysis was performed using Ion Reporter™ Software v5.18.4.0 (ThermoFisher Scientific). Chimeric sequences were automatically identified and removed. Reads were mapped to reference databases Greengenes v13.5 and MicroSEQ ID v3.0. Gut microbiome profiles were compiled using metagenome taxonomic data *via* the Curated MicroSEQ(R) 16s Reference Library v2013.1. Raw abundance values were subsampled at 10,000 reads per sample to control for inequivalent read numbers across samples. Subsampling was performed on the species-level operational taxonomic unit (OTU) table *via* the rrarefy function of the vegan R package (Oksanen et al., 2022). Samples with less than 10,000 total reads were excluded from the dataset. Upstream taxonomic ranks were determined by systematically comparing family-level OTU data to the NCBI database *via* the classification function of the taxize R package (Chamberlain et al., 2020). Family-level OTU table was the preferred classification input due to large amounts of unclassified upstream classifications when using genus and species-level OTU tables. Genus and species-level OTU tables were joined onto the family-OTU table to form a comprehensive taxonomic classification. Subsampled reads on the species-level were converted to per-sample relative abundance values *via* the “transform” function of the microbiome R package (Lahti and Shetty, 2022). α-diversity values according to Shannon, Simpson and Chao-1 indices were computed *via* IonReporter v5.18.4.0.

### PBMC specimens, monocyte enrichment, and nucleic acid isolation

Viable cryopreserved PBMCs of approximately 1 × 10^7^ from individuals were first thawed in AIM-V Serum Free Media (Thermo Fisher Scientific, Inc., Waltham, MA, United States) supplemented with 1:50 DNase (Sigma-Aldrich, St. Louis, MO, United States), washed, and resuspended in wash buffer (PBS, 3% BSA, and 1 mM EDTA). Aliquots of 1.25 × 10^5^ cells (PBMCs) were taken from each sample before enrichment for flow cytometry-based cellular phenotyping assays to determine cell type composition. Monocytes were enriched from PBMCs using the Negative-Selection, Human Monocyte Enrichment Kit without CD16 Depletion (StemCell Technologies, Inc., Vancouver, BC, Canada) following the manufacturer’s guidelines utilizing the purple EasySepTM magnet (StemCell Technologies). Cells were counted before cell separation using the Countess^®^ Automated Cell Counter (Life TechnologiesTM, Carlsbad, CA, United States) to determine the minimal cell concentration required for cell: antibody/magnetic bead binding for effective monocyte enrichment. Negatively-selected cells were counted again after monocyte enrichment to partition the appropriate number of cells required for flow cytometry (1.0 × 10^5^–1.25 × 10^5^ cells) to confirm the efficiency of monocyte enrichment, while the remainder of enriched cells were pelleted and resuspended in lysis buffer for subsequent purification of nucleic acids. DNA and RNA were isolated from enriched monocytes using the MagMAX FFPE DNA/RNA Ultra Kit (Thermo Fisher Scientific) according to manufacturer recommendations for animal cell applications. Nucleic acid concentrations were quantified using the Qubit^®^ 2.0 Fluorometer (Thermo Fisher Scientific) following manufacturer protocols. Qubit^®^ dsDNA HS Assay Kit and Qubit^®^ RNA BR Assay Kit (Thermo Fisher Scientific) were used for DNA and RNA, respectively. DNA and RNA were stored at −20°C and −80°C, respectively.

### Validation of monocyte enrichment by flow cytometry

To confirm the enrichment of monocytes by negative selection, an aliquot of PBMCs (1.25 × 10^5^ cells) for all subjects pre- and post-enrichment was analyzed by a flow cytometer for monocytes, T cells, NK cells, and B cells. Aliquots were stained with yellow amine fluorescent reactive dye (YARD; Thermo Fisher Scientific), then with anti-CD16 Brilliant Violet 421 (Clone 3G8), anti-CD3 V500 (Clone UCHT1), anti-CD14 Qdot^®^605 (Clone TüK4), anti-CD56 Pe-Cy7 (Clone B159), anti-CD19 PE-Cy7 (Clone SJ25C1), anti-CD20 Pe-Cy7 (Clone 2H7), and anti-HLA-DR APC-H7 (Clone G46-6) for identification of leukocyte subpopulation frequencies. Anti-CD16 was purchased from BioLegend, Inc., San Diego, CA, United States. Anti-CD3, anti-CD56, anti- CD20, anti-CD19, and anti-HLA-DR were obtained from BD Bioscience, San Jose, CA, United States. Anti-mouse Ig/Negative Control (FBS) Compensation Particle Set (BD Bioscience) was used for compensation analysis of fluorescent signals emitted by each fluorochrome from the multi-colored cellular phenotyping panel employed. Anti-mouse Ig compensation beads were stained with each fluorochrome-conjugated antibody in separate wells. ArCTM Amine Reactive Compensation Bead Kit (ThermoFisher Scientific) reactive bead/negative beads were used for compensation of YARD (Live/Dead stain) fluorescent signals. Stained cells from PBMCs, enriched monocytes and compensation particles were analyzed using a 4-laser BD LSRFortessa flow cytometer (BD Bioscience). The data was analyzed using the FlowJo software (Tree Star, Inc., Ashland, OR, United States). The frequency (%) of each cell type was determined by event count (specific event/total events) with debris exclusion. Successful enrichment was observed for all samples in each group with an average monocyte enrichment of at least 90%. High-quality enrichment was necessary to diminish background noise caused by mixed cell populations, as the heterogeneity of cell populations confounds downstream DNA methylation analyses.

### Illumina EPIC array-based DNA methylation analysis

The Illumina Infinium MethylationEPIC BeadChip (Illumina^®^, Inc., San Diego, CA, United States) is capable of quantifying DNA methylation at over 850,000 cytosine-guanine dinucleotides (CpG) distributed throughout the genome at single-nucleotide resolution based on the ratio of fluorescent intensities between the methylated and unmethylated alleles of each CpG locus. To perform the EPIC microarray using the Illumina^®^ Infinium^®^ HD Methylation assay (Illumina^®^, Inc.), 500 ng of genomic DNA from isolated monocytes was first treated with sodium bisulfite using the EZ DNA MethylationTM Kit (Zymo Research, Irvine CA, United States) following manufacturer protocols. Illumina iScan SQ scanner was utilized for chip imaging to receive intensities of hybridized CpG probes.

Raw IDAT files were analyzed using R statistical software (v4.1.2) *via* the RnBeads package (v2.12.2) (Mueller et al., 2019; Yassen et al., 2014). RnBeads is a start-to-finish pipeline for DNA methylation analysis in accordance with previously established standards and practices (Bock, 2012; Michels et al., 2013; Wreczycka et al., 2017). The package also implements multiple normalization methods available in several other software packages, such as minfi and Watermelon (Xu et al., 2021). RnBeads is a widely used tool for DNA methylation analysis (200–300 downloads per month from Bioconductor) and has been referenced in multiple articles (Müller et al., 2019). In our case, an RnBeads-centric approach was utilized due to its ability to pre-process raw data files, perform differential analysis and output β (beta) value matrices within a single function, minimizing the risk of administrative error. CpG-specific methylation (β values; 0.00–1.00, as a percent from unmethylated to methylated) was determined following multiple pre-processing steps within the RnBeads framework. Low quality probes and samples were removed *via* the greedycut algorithm, which iteratively produces subsets of the β matrix until sample and/or probe removal is no longer necessary to ensure a uniform detection *p*-value ≥0.05. One of the generated β matrices is chosen as the most reliable and undergoes subsequent analyses. The optimal β matrix is the one that provides the maximum value of the expression *s + 1* – *a*, where (a) denotes false positive rate and (s) denotes sensitivity. Potential batch effect bias was controlled *via* per-Sentrix ID and position normalization. Such normalization accounts for the spatial variance of samples on the physical array. Background correction was performed using normal-exponential out-of-band correction *via* the methylumi package (Davis et al., 2022). Functional normalization was performed *via* the minfi package (Maksimovic et al., 2012; Triche et al., 2013; Aryee et al., 2014; Fortin et al., 2014; Andrews et al., 2016; Fortin et al., 2016).

Epigenetic signatures were compared across SE groups *via* the identification of differentially methylated loci (DML) *via* Epic Array. β-values were compared *via* Welch’s *t*-test, which is the default statistical test utilized by the RnBeads framework. Variables such as age, A1c (%), body mass index and sex were not defined as covariates during differential analysis, given that said variables did not differ significantly across SE groups (Table 1). CpG sites were further evaluated *via* Empirical Bayes permutation *t*-test between NSE and LSE groups. Resultant Permutation *p*-values were adjusted for potential multiple comparisons errors *via* the Benjamini–Hochberg method. Gencode annotations were obtained *via* the Illumina Epic Array Manifest file (B5, v1.0). Permutation tests were performed at 1,000 iterations *via* the RBM_T function of the RBM R package (v1.26.0) with statistical significance determined at α = 0.05 (Li and Liang, 2021). Such an approach provides higher statistical power and reduces false discovery rates for non-normally distributed array-based data. DML β-value distribution and ∆β values were visualized *via* the ComplexHeatmap R package (v 2.12.1) (Gu et al., 2016; Gu, 2022). Hierarchical clustering of participants and DML were performed using Euclidean distances. DML were evaluated for pathway enrichment in accordance with NCBI gene ontology and KEGG databases, *via* the *gometh* function of the missmethyl R package (Phipson et al., 2016). Separate database searches were performed with promoter and gene-body exclusive DML. DML were ranked according to permutation ∆β values to determine the top ten most differentially methylated loci. β-values for these DML were evaluated individually for effects upon nominal SE score *via* linear regression.

**TABLE 1:** Sociodemographic and biometric summary of our NHPI-enriched cohort stratified by self-esteem score. Biometric summary of our cohort displaying comparative and correlational analyses with respect to self-esteem score.

	Total cohort	SE groups			SE score correlation	
		LSE	NSE	P	R^c^	P
Participants (N; %)	68	23 (34%)	45 (66%)			
Sex (N; %)				0.914^a^		
Female	42 (62%)	14 (61%)	28 (62%)			
Male	26 (38%)	9 (39%)	17 (38%)			
Age (mean ± SEM)	38 ± 2	42 ± 4	35 ± 3	0.059^b^	−0.25	**0.039**
SE Score	17.07 ± 0.31	14.48 ± 0.21	18.40 ± 0.31	**<0.001** **^b^**		
A1C (mean ± SEM)	5.84 ± 0.22	5.68 ± 0.31	5.92 ± 0.29	0.948^b^	−0.06	0.623
T2DM categories (N; %)				0.228^a^		
Non-diabetic	50 (73%)	15 (65%)	35 (78%)			
Pre-diabetic	4 (6%)	3 (13%)	1 (2%)			
Diabetic	14 (21%)	5 (22%)	9 (20%)			
BMI (mean ± SEM)	32 ± 1	34 ± 2	31 ± 2	0.133^b^	−0.22	0.074
BMI categories (N; %)				0.287^a^		
Normal	15 (22%)	4 (17%)	11 (24%)			
Overweight	8 (12%)	1 (4.3%)	7 (16%)			
Obese	45 (66%)	18 (78%)	27 (60%)			
Ethnicity (N; %)				0.619^a^		
NHPI	47 (69%)	15 (65%)	32 (71%)			
non-NHPI^d^	21 (31%)	8 (35%)	13 (29%)			

Bold *p*-values indicate statistical significance at α = 0.05.

^a^ Fisher’s exact test.

^b^ Mann-Whitney U test with Bonferroni-Dunn *p*-value adjustment.

^c^ Spearman correlation coefficient.

^d^ Individuals classified as “non-NHPI” include those who self-identified as Asian (7.35%), Non-Hispanic White People (5.88%), and other non-NHPI, participants with undisclosed racial-ethnic data (17.65%).

### Epigenetic clock analyses

DNA methylation data measured by the MethylationEPIC Array was evaluated for potential molecular indicators of epigenetic age (Epi-Age) acceleration. Participant age predictions were calculated using the *methylclock* package (Pelegí-Sisó et al., 2021) within the R statistical software. Methylation-based age predictions were obtained *via* the Horvath (Horvath, 2013), Hannum (Hannum et al., 2013) and Levine (PhenoAge) (Levine et al., 2018) predictive models. Results of Epi-Age acceleration analyses are reported as the difference between the predicted age from each respective model and chronological age. Positive values are indicative of Epi-Age acceleration. Negative values are indicative of Epi-Age deceleration.

### Statistical analyses

Comparative analyses of clinical and NGS data were performed using the Mann-Whitney U test. Non-parametric tests for clinical and NGS data to fit non-normal data distribution (confirmed using the D’Agostino and Pearson test for normality). To predict associative outcomes, several stepwise multiple logistic regression models were tested on clinical features, and NGS results were identified by Mann-Whitney U tests followed by multiple testing corrections (FDR-adjusted *p < 0.01*). Graphing and statistical analysis were performed using Prism 9, Version 9.0c (GraphPad, La Jolla, CA, United States). All statistical significance was determined at *p < 0.05*. Outliers falling beyond the 1.5 IQR range were omitted.

Any immunometabolic hormone quantitation and relative abundance of gut bacterial taxa that significantly correlated with SE score were selected as independent variables in linear regression analysis. Linear regression was performed using the R package (v. 4.2.1) (R Core Team, 2022). SE scores were log-linear transformed to report the association effects as percentages. All statistical significance was determined at *p <* 0.05 with Bonferroni correction where appropriate. Additionally, for linear regression analysis, a dummy interaction to the selected relative abundance of gut bacterial taxa were used to control for the samples with zero selected relative abundance value.

## Results

### Sociodemographic and biometric summary of NHPI-enriched cohort

**FIGURE 2:**
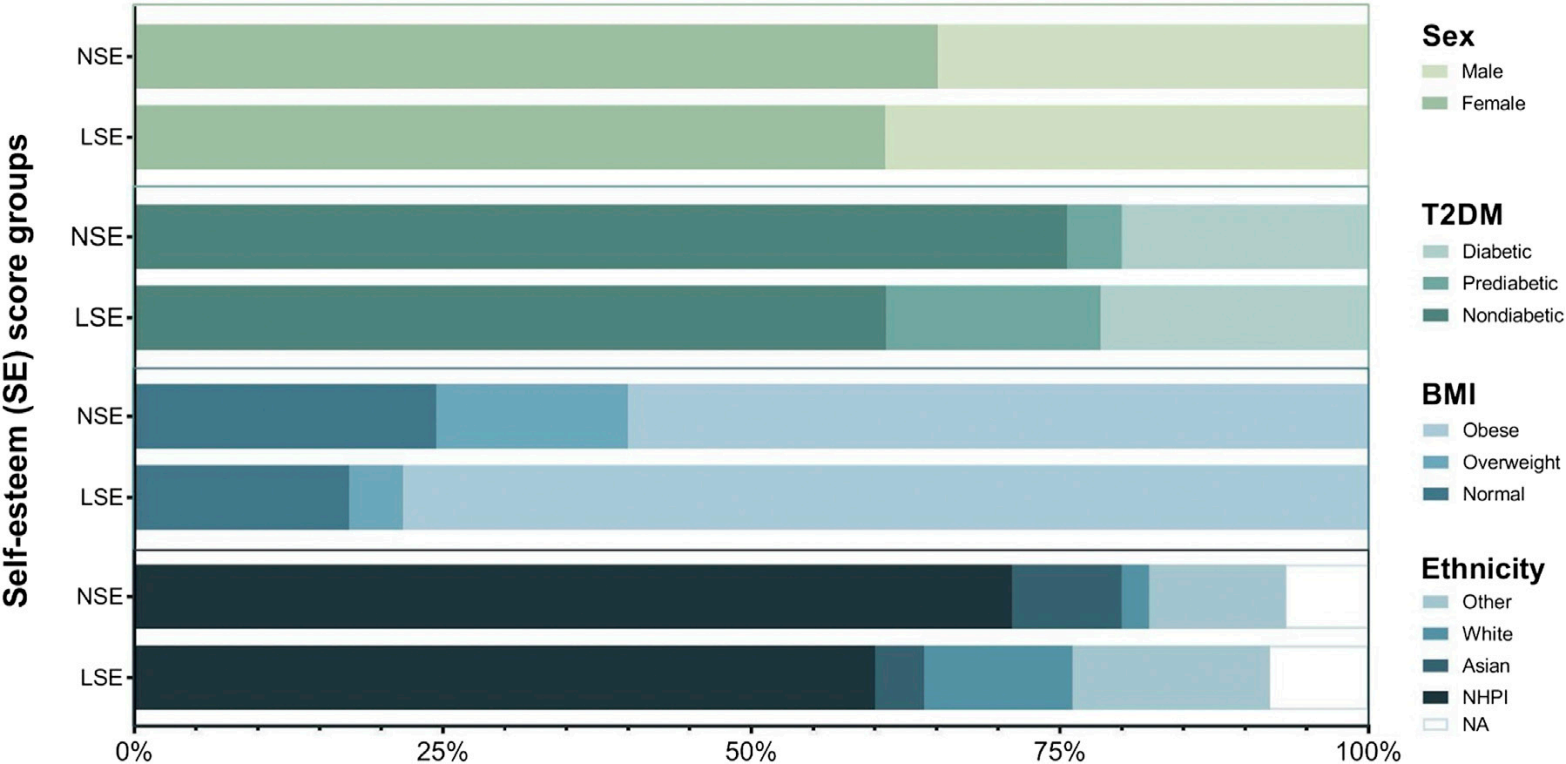
Graphical summary of biometric and sociodemographic category distribution across SE groups. Chi-squared tests were performed between SE groups to assess the independence of categorical distribution for sex (*p* = 0.914), T2DM (*p* = 0.228), BMI (*p* = 0.287), and ethnicity (*p* = 0.619).

A summary of sociodemographic data within our NHPI-enriched cohort is presented in Table 1. 69% of participants self-identified as NHPI (*N* = 47). Participants age ranged from 17 to 79 years, with an average of 38 years, and were predominantly female (62%). Survey scores qualified 66% of participants with NSE (*N* = 45) and the remaining 33.8% with LSE (*N* = 23). The relationships between the SE score groups and sociodemographic categories of interest were statistically insignificant. As illustrated in Figure 2, the categorical distributions of sex, T2DM risk, BMI, and ethnicity across the SE groups were also statistically insignificant, altogether indicating that these factors are unlikely covariates of SE in this cohort.

### Immunometabolic biomarkers in the context of self-esteem

Plama levels of immunometabolic biomarkers and comparative immune cell profiling upon SE stratification are summarized in Table 2. Compared to NSE individuals, LSE individuals exhibited lower concentrations of immunometabolic hormone adiponectin (*p =* 0.004). Furthermore, adiponectin bioavailability correlated positively with SE scores (*R* = 0.34*; p* = 0.013)*.* This trend suggests that LSE may exacerbate NHPI predisposition to T2DM by way of reduced bioavailability of adiponectin. As adiponectin is known to play a role in insulin signaling and inflammatory pathways (Ouchi and Walsh, 2007), its reduction may be partially responsible for the onset of insulin resistance and systemic inflammation and T2DM pathophysiology. This notion is reinforced by the observed negative correlation between SE score and circulating insulin (*R* = −0.39*; p* = 0.004), indicating glycemic dysregulation with decreasing SE score.

**TABLE 2:** Intergroup comparison and correlational analyses of immunometabolic biomarker concentrations by SE.

	Self-esteem (SE) groups			SE correlation	
	NSE	LSE	*P* ^a^	*R* ^b^	*P*
Immunometabolic hormone quantitation (mean ± SEM)					
Adiponectin (μg/mL)	3.45 ± 0.34	1.68 ± 0.18	**0.004**	0.34	**0.013**
Leptin (ng/mL)	10.90 ± 1.14	11.37 ± 1.23	0.259	−0.12	0.344
Pyy (pg/mL)	72.73 ± 5.84	88.23 ± 22.74	0.774	0.12	0.404
Cortisol (ng/mL)	25.84 ± 2.26	21.09 ± 2.89	0.272	0.22	0.079
Insulin (μIU/mL)	3.13 ± 0.11	3.72 ± 0.22	**0.014**	−0.39	**0.004**
Cytokine and other biomarker quantitation (mean ± SEM)					
TNF-α (pg/mL)	1.62 ± 0.28	2.49 ± 0.39	**0.011**	−0.27	**0.031**
IL-6 (pg/mL)	2.12 ± 0.38	4.47 ± 1.28	0.075	−0.26	**0.044**
IFN-γ (pg/mL)	1.42 ± 0.22	1.83 ± 0.33	0.295	−0.15	0.253
IL-10 (pg/mL)	3.67 ± 0.50	3.38 ± 0.63	0.823	0.09	0.484
IL-1β (pg/mL)	4.60 ± 0.52	6.13 ± 0.86	0.129	−0.10	0.439
IL-8 (pg/mL)	22.65 ± 1.85	31.03 ± 3.59	**0.051**	−0.17	0.176
GLP-1*3 (pg/mL)	43.54 ± 3.05	38.24 ± 4.78	0.613	0.06	0.655
CRP (ng/mL)	141.15 ± 27.73	162.09 ± 48.53	0.553	−0.19	0.142
MCP-1 (pg/mL)	234.17 ± 24.73	309.80 ± 48.47	0.305	−0.09	0.483
VEGF-A (pg/mL)	254.35 ± 36.20	311.48 ± 52.19	0.307	−0.21	0.098

Bold *p*-values indicate statistical significance at α = 0.05.

^a^ Mann-Whitney *U* tests.

^b^ Spearman correlation coefficient.

Conversely, proinflammatory cytokine concentration of TNF-α (*p* = 0.011) and chemokine concentration of IL-8 (*p* = 0.051) were higher in the LSE relative to the NSE group. TNF-α and IL-8 play important roles in adipose tissue metabolism and function and have been linked to various inflammatory diseases (Bruun et al., 2001; Popko et al., 2010). Indeed, depression is characterized by an increase in levels of various proinflammatory factors, and LSE has been found to be a good predictor for depression later in life. Thus, our observation of TNF-α and IL-8 being higher in the LSE group is consistent with this previous finding.

While IL-6 levels did not significantly differ between SE groups, it did exhibit a significantly negative correlation with SE score (*R* = −0.26; *p* = 0.044). As chronic exposure to high levels of IL-6 has been found to mechanistically induce insulin resistance by upregulating SOC-3 expression (an inhibitor of insulin signaling) and impair insulin receptor signal transduction (Rehman and Akash, 2017), these results suggest that LSE may exacerbate immunometabolic dysregulation *via* elevated levels of IL-6, and downstream pathophysiological effects resembling T2DM progression.

### Metagenomic analysis of the gut microbiome

**FIGURE 3:**
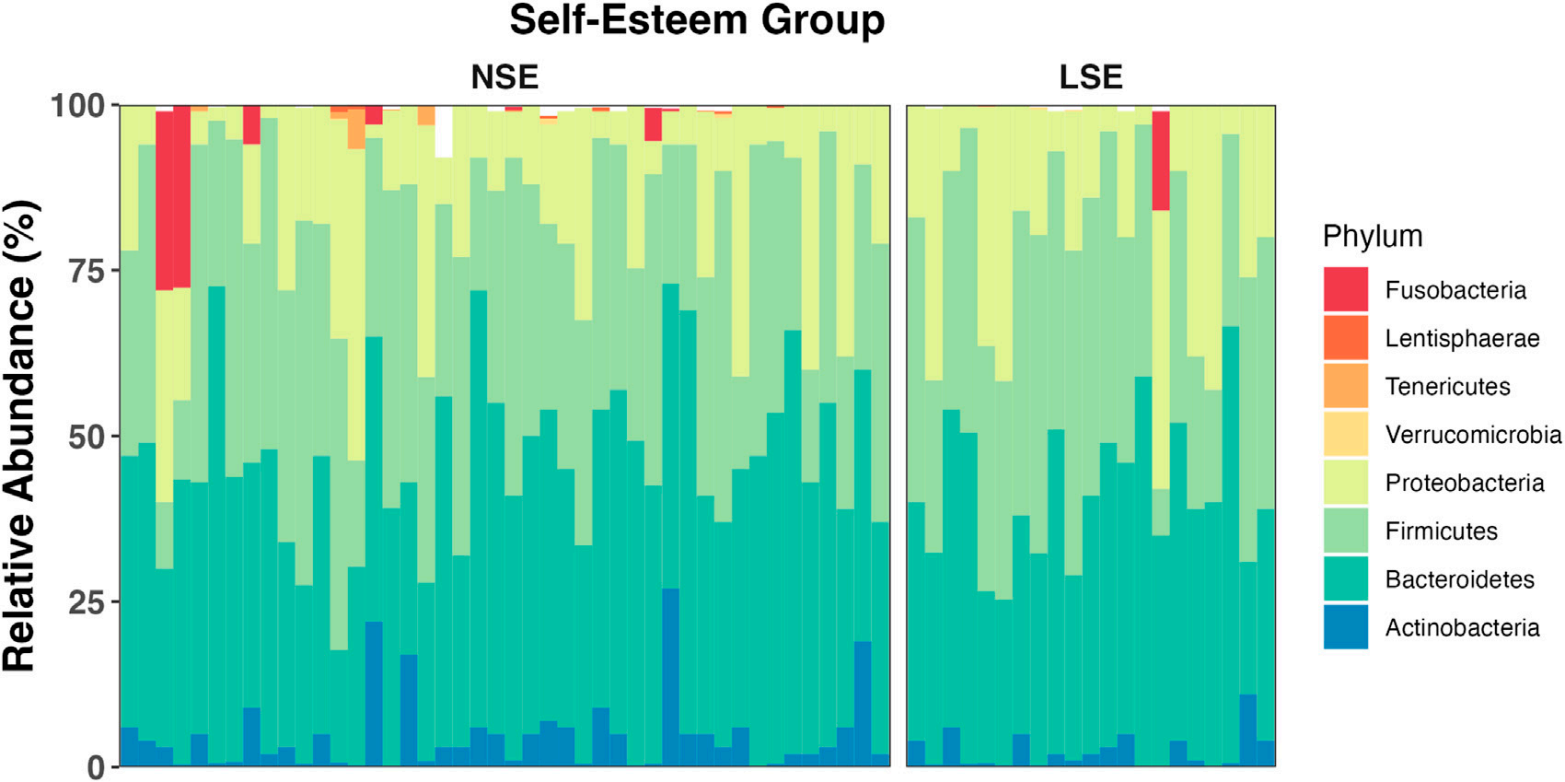
Average relative abundance of gut bacterial phyla across SE groups. Bacterial phyla *Bacteroidetes*, *Firmicutes*, *Proteobacteria*, and *Actinobacteria* (among unclassified bacteria) compose an average of roughly 97% of the gut bacterial population in each group. The remaining 3% of the gut bacteria are largely composed of members belonging to *Verrucomicrobia*, *Tenericutes*, *Lentisphaerae*, and *Fusobacteria*. The mean relative abundance of bacterial phyla is represented for NSE and LSE groups, and for each individual participant.

Relationships between gut bacterial taxa and SE are summarized in Table 3. A graphical summary of phylum-level gut microbiome composition is provided in Figure 3 for each participant upon SE stratification. Quality control data summarizing metagenomic sequencing are provided in Supplementary Table S3.

**TABLE 3:** Intergroup comparisons and linear regression analyses for the mean relative abundance of identified gut bacterial taxa at the family, genus, and species levels with respect to SE.

	Self-esteem (SE) groups			SE linear regression	
	NSE	LSE	*P* ^a^	*R* ^2^	*P*
Family-level relative abundance (%; mean ± SEM)					
*Coriobacteriaceae*	3.25E-02 ± 7.87E-03	9.67E-03 ± 2.03E-03	**0.014**	0.26	**0.034**
*Acholeplasmataceae*	0.00E+00 ± 0.00E+00	1.15E-03 ± 8.64E-04	**0.015**	−0.26	**0.033**
*Clostridiales* *^b^*	1.24E-04 ± 4.50E-05	0.00E+00 ± 0.00E+00	**0.016**	0.38	**0.002**
*Oxalobacteraceae*	6.96E-04 ± 2.25E-04	8.00E-03 ± 6.59E-03	**0.029**	−0.32	**0.008**
*Brucellaceae*	1.47E-04 ± 1.44E-04	1.80E-03 ± 1.30E-03	**0.025**	−0.18	0.134
*Rhizobiaceae*	7.33E-05 ± 7.33E-05	1.23E-03 ± 7.65E-04	**0.025**	−0.19	0.127
*Gracilibacteraceae*	0.00E+00 ± 0.00E+00	1.83E-04 ± 1.69E-04	**0.049**	−0.23	0.054
*Pseudanabaenaceae*	0.00E+00 ± 0.00E+00	9.67E-03 ± 6.38E-05	**0.015**	−0.21	0.085
*Verrucomicrobiaceae*	1.04E-04 ± 5.07E-05	2.65E-04 ± 1.45E-04	0.132	−0.31	**0.010**
*Veillonellaceae*	2.68E-02 ± 3.92E-03	2.37E-02 ± 1.17E-02	0.061	0.28	**0.022**
*Syntrophomonadaceae*	1.13E-04 ± 7.92E-05	4.17E-04 ± 2.94E-04	0.089	−0.25	**0.043**
Genus-level relative abundance (%; mean ± SEM)					
*Mitsuokella*	1.29E-03 ± 5.70E-04	8.39E-04 ± 4.66E-04	**0.016**	0.29	**0.015**
*Collinsella*	8.12E-03 ± 2.63E-03	6.59E-03 ± 2.80E-03	**0.020**	0.27	**0.023**
*Herbaspirillum*	3.87E-03 ± 3.14E-03	8.52E-04 ± 3.14E-04	**0.034**	−0.28	**0.021**
*Cloacibacillus*	1.67E-04 ± 1.67E-04	8.26E-05 ± 8.26E-05	**0.049**	−0.17	0.165
*Akkermansia*	2.44E-04 ± 2.44E-04	1.35E-04 ± 9.15E-05	0.124	−0.31	**0.009**
*Lachnoclostridium*	2.28E-03 ± 6.04E-04	2.32E-03 ± 6.17E-04	**0.051**	−0.27	**0.028**
*Megasphaera*	1.70E-03 ± 7.68E-04	4.36E-03 ± 1.68E-03	0.261	0.24	**0.046**
Species-level relative abundance (%; mean ± SEM)					
*Blautia faecis*	3.56E-05 ± 1.86E-05	1.48E-04 ± 5.58E-05	**0.007**	−0.25	**0.040**
*Lachnoclostridium clostridioforme*	1.33E-03 ± 3.89E-04	2.98E-03 ± 7.99E-04	**0.007**	−0.34	**0.004**
*Alistipes* sp.	3.56E-04 ± 1.72E-04	1.15E-03 ± 4.05E-04	**0.012**	−0.32	**0.009**
*Mitsuokella jalaludinii*	9.47E-04 ± 3.48E-04	0.00E+00 ± 0.00E+00	**0.016**	0.30	**0.014**
*Mitsuokella multacida*	4.67E-04 ± 1.93E-04	0.00E+00 ± 0.00E+00	**0.016**	0.30	**0.014**
*Bacteroides intestinalis*	7.80E-04 ± 4.05E-04	9.13E-04 ± 3.60E-04	**0.021**	−0.27	**0.024**
*Collinsella aerofaciens*	9.83E-03 ± 2.86E-03	2.61E-03 ± 7.34E-04	**0.030**	0.27	**0.028**
*Bulleidia p.1630.c5*	4.49E-04 ± 2.00E-04	0.00E+00 ± 0.00E+00	**0.049**	0.28	**0.020**
*Bacteroides cellulosilyticus*	3.09E-04 ± 2.87E-04	7.35E-04 ± 4.06E-04	**0.023**	−0.20	0.094
*Eubacterium eligens*	6.83E-03 ± 2.76E-03	1.23E-02 ± 3.73E-03	**0.030**	−0.09	0.459
*Parabacteroides goldsteinii*	1.11E-03 ± 1.10E-03	3.91E-04 ± 2.68E-04	**0.030**	−0.21	0.087
*Alistipes indistinctus*	3.33E-04 ± 1.63E-04	0.00E+00 ± 0.00E+00	**0.034**	0.19	0.117
*Roseburia faecis*	1.40E-02 ± 2.43E-03	2.21E-02 ± 3.76E-03	**0.040**	−0.19	0.119
*Bacteroides caccae*	3.83E-03 ± 1.58E-03	5.37E-03 ± 1.32E-03	**0.044**	−0.22	0.078
*Lachnobacterium* sp.	0.00E+00 ± 0.00E+00	2.17E-04 ± 2.08E-04	**0.049**	−0.17	0.165
*Sutterella parvirubra*	0.00E+00 ± 0.00E+00	3.91E-05 ± 2.72E-05	**0.049**	−0.24	0.052
*Parabacteroides johnsonii*	0.00E+00 ± 0.00E+00	4.30E-04 ± 3.32E-04	**0.049**	−0.22	0.077
*Cloacibacillus evryensis*	0.00E+00 ± 0.00E+00	3.52E-04 ± 3.26E-04	**0.049**	−0.17	0.165
*Desulfovibrio* sp.	0.00E+00 ± 0.00E+00	4.35E-05 ± 3.26E-05	**0.049**	−0.23	0.060
*Akkermansia muciniphila*	1.51E-04 ± 7.31E-05	2.83E-04 ± 1.44E-04	0.223	−0.28	**0.020**
*Megasphaera hominis*	2.36E-03 ± 8.18E-04	9.22E-04 ± 5.30E-04	0.369	0.26	**0.034**
*Lactobacillus mucosae*	9.56E-05 ± 8.30E-05	0.00E+00 ± 0.00E+00	0.319	0.26	**0.035**

Bold *p*-values indicate statistical significance at α = 0.05.

^a^ Mann-Whitney U test (unpaired, two-tailed).

^b^ *Family.XIII. Incertae.Sedis*.

Compared to those for individuals with NSE, relative abundance values for gut bacterial families *Coriobacteriaceae* (*p* = 0.014) and *Clostridiales.Family.XIII.Incertae.Sedis* (*p* = 0.016) were significantly higher in LSE individuals. Additionally, the respective relative abundances of these families were positively associated with SE score (*Coriobacteriaceae: R* = 0.26; *p* = 0.034; *Clostridiales.Family.XIII.Incertae.Sedis: R* = 0.38; *p* = 0.002). The opposite trend was observed after intergroup comparison for families *Acholeplasmataceae* (*p* = 0.015), *Oxalobacteraceae* (*p* = 0.029), *Brucellaceae (p* = 0.025), *Rhizobiaceae (p* = 0.025), *Gracilibacteraceae (p* = 0.049), and *Pseudanabaenaceae (p* = 0.015). Of these taxa, *Acholeplasmataceae* (*R* = −0.26; *p* = 0.033) and *Oxalobacteraceae* (*R* = −0.32; *p* = 0.008) were significantly negatively associated with SE score. Although SE score demonstrated additional significant relationships with *Veillonellaceae* (*R* = 0.28; *p* = 0.022), *Verrucomicrobiaceae (R* = −0.31; *p* = 0.010), and *Syntrophomonadaceae* (*R* = −0.25; *p* = 0.043), sample distribution among their respective relative abundance values did not significantly differ between SE groups.

Genera that were differentially abundant between SE groups included *Mitsuokella* (*p* = 0.016), *Collinsella* (*p* = 0.020), *Herbaspirillum* (*p* = 0.034), *Cloacibacillus* (*p* = 0.049), and *Lachnoclostridium* (*p* = 0.051). Of these genera associations with SE score were observed to be significantly positive for *Mitsuokella* (*R* = 0.29; *p* = 0.015) and *Collinsella* (*R* = 0.27; *p* = 0.023), and negative for *Herbaspirillum* (*R* = −0.28; *p* = 0.021) and *Lachnoclostridium* (*R* = −0.27; *p* = 0.028).

At the species level, the relative abundance of *Lachnoclostridium clostridioforme* (*p* = 0.007), *Alistipes* sp. (*p* = 0.012), *Bacteroides intestinalis* (*p* = 0.021), *Blautia faecis* (*p* = 0.007), *Sutterella parvirubra* (*p* = 0.049), *Desulfovibrio* sp. (*p = 0.049*), *Bacteroides caccae* (*p* = 0.044), *Parabacteroides johnsonii* (*p* = 0.049), *Parabacteroides goldsteinii* (*p* = 0.030), *Bacteroides cellulosilyticus* (*p* = 0.023), *Roseburia faecis* (*p* = 0.040), *Lachnobacterium* sp. (*p* = 0.049), *Cloacibacillus evryensis* (*p* = 0.049), and *Eubacterium eligens* (*p* = 0.030) were significantly higher in the LSE group, whereas *Alistipes indistinctus* (*p* = 0.034), *Collinsella aerofaciens* (*p* = 0.030), *Bulleidia p.1630.c5* (*p* = 0.049), *Mitsuokella jalaludinii* (*p* = 0.016), and *Mitsuokella multacida* (*p* = 0.016) were significantly lower. Of these species, *C. aerofaciens* (*R* = 0.27; *p* = 0.028), *Bulleidia p.1630.c5* (*R* = 0.28*; p* = 0.020), *M. jalaludinii* (*R* = 0.30; *p* = 0.014), and *M. multacida* (*R* = 0.30; *p* = 0.014) exhibited a significant positive correlation with SE score, whereas *L. clostridioforme* (*R* = −0.34; *p* = 0.004), *Alistipes* sp. (*R* = −0.32; *p* = 0.009), *Bacteroides intestinalis* (*R =* −*0.27; p* = 0.024), and *B. faecis* (*R* = −0.24; *p* = 0.040) negatively correlated with SE score. No significant trends were observed for gut microbial α-diversity in the context of SE (Supplementary Table S2).

### Monocyte methylation states and epigenetic clock analysis as an indicator of LSE

**FIGURE 4:**
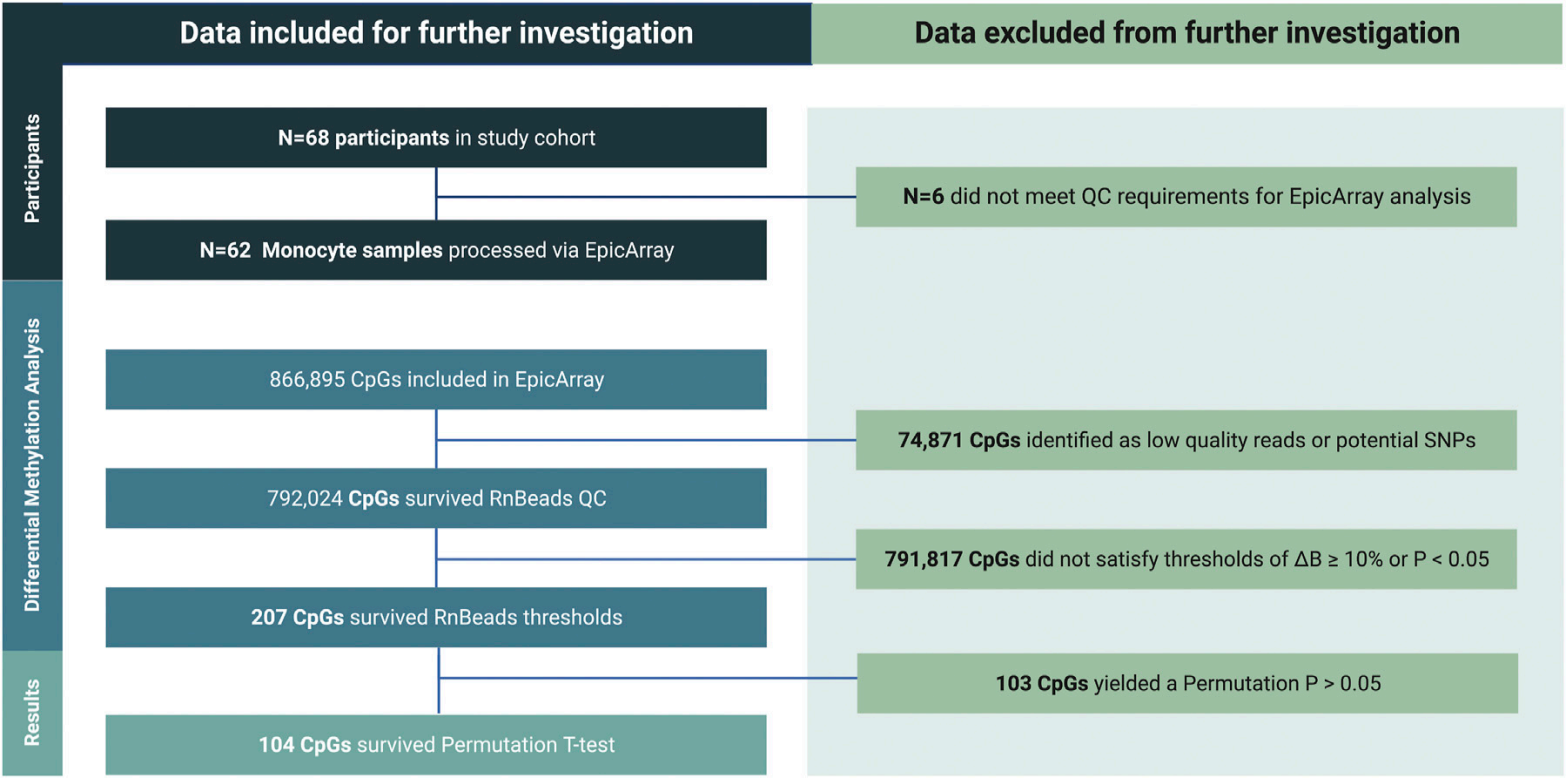
Inclusion-exclusion criteria utilized to determine the final set of differentially methylated loci (DML) upon SE stratification for downstream analyses. Of the ∼850,000 CpG sites considered on the standard Infinium MethylationEPIC Array, those possessing a mean ∆β of <10% between NSE and LSE groups and/or a *p*-value of >0.05 during RnBeads differential methylation analysis were eliminated for consideration among differentially methylated loci (DML). Surviving CpG sites were further evaluated *via* Empirical Bayes permutation *t*-test between NSE and LSE groups.

Inclusion-exclusion criteria of DML associated with SE are summarized in Figure 4.

Methylation patterns among DML exhibiting a mean Δβ-value of >10% upon SE stratification are graphically represented in Supplementary Figure S1A. DML were significantly hypomethylated in LSE relative to NSE (*p* < 0.001), with β-values of 0.80, 0.40, and 0.10 being the most frequently observed in both SE groups (Supplementary Figure S1B). The majority of the DML (*N* = 51; 49%) were located in intergenic regions, most (*N* = 34; 67%) of which were hypermethylated with respect to LSE. Significant differences between the number of DML hypo- and hyper-methylated were found predominantly at 5′UTR and intergenic regions (Supplementary Figure S1C), potentially indicating functional differences in transcription of associated genes.

**FIGURE 5:**
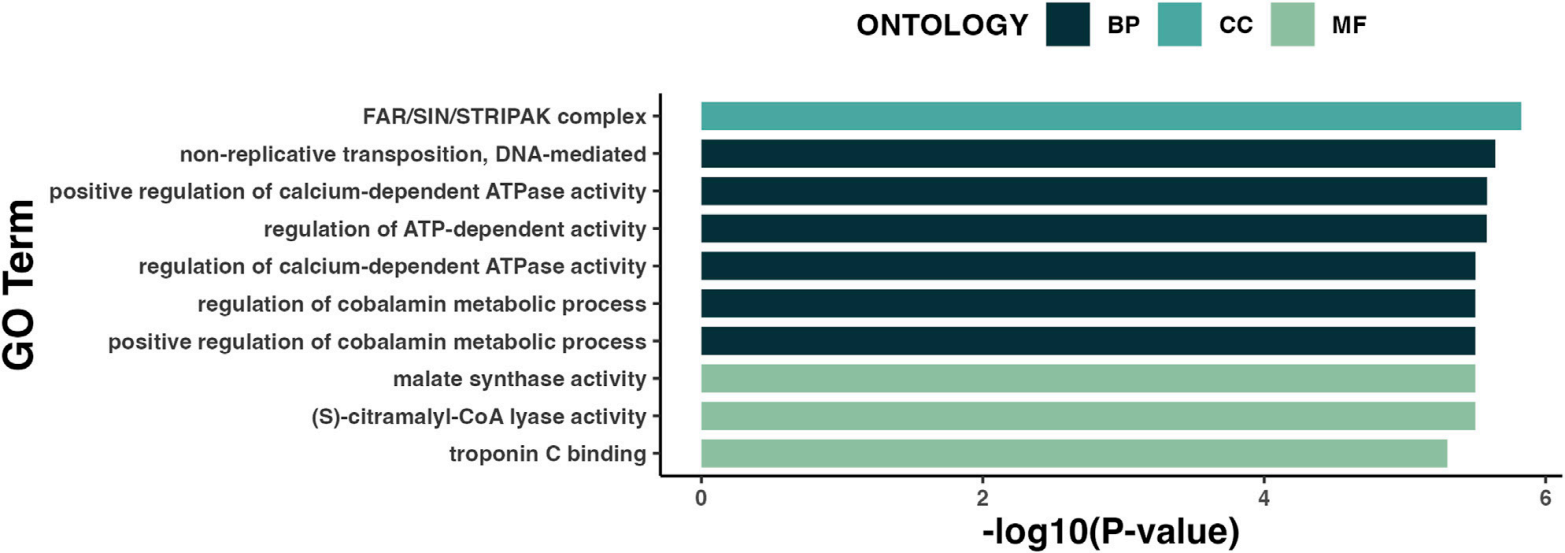
Gene-ontology (GO) pathway analysis of DML associated with SE. Results are represented in order of enrichment score [-log (*p*-value)]. BP, Biological process; CC, Cellular component; MF, Molecular function.

Gene-ontology (GO) pathway analysis of all DML by SE showed enrichment of genes involved in biological processes related to metabolic mechanisms (Figure 5). Existing literature has described an association between LSE and obesity, implicating metabolic dysregulation as a link between the two (Moradi et al., 2022). Although BMI and A1c did not significantly differ between the SE groups (Table 1), plasma levels of insulin were significantly higher (*p = 0.014*) in individuals with LSE (Table 2).

The top ten most differentially methylated loci (DML) were further evaluated for potential effects on nominal SE scores *via* linear regression (Table 4). The associated gene for each DML was also obtained *via* the manifest file. For six of these DML, mean β-values were significantly associated with SE: cg12914114 (*p* = 0.028), cg25436480 (*p* = 0.021), cg01695532 (*p* = 0.007), cg18232235 (*p* = 0.026), cg04402345 (*p* = 0.015), and cg19288863 (*p* = 0.004). These linear regressions are graphically summarized in Supplementary Figure S2.

**TABLE 4:** Intergroup comparisons and linear regression between SE scores and respective β-values of 10 most differentially methylated loci (DML).

				β-values (mean ± SEM)			SE linear regression	
	Gene(s)	Region	CpG	NSE	LSE	*p* ^a^	Coeff.	*p*
cg11956442	UGDH-AS1	5′ UTR	Shelf	0.29 ± 0.05	0.56 ± 0.06	**0.002**	−1.50	0.139
cg03600007	CACNG3	Intergenic	—	0.45 ± 0.04	0.68 ± 0.04	**0.002**	−1.42	0.249
cg12914114	FAM120B	Intergenic	Island	0.33 ± 0.04	0.55 ± 0.07	**0.024**	−2.30	**0.028**
cg25436480	ABP1	Promoter	—	0.25 ± 0.04	0.47 ± 0.06	**0.002**	−2.70	**0.021**
cg01695532	STRN	Intergenic	Shelf	0.86 ± 0.03	0.64 ± 0.06	**0.017**	3.50	**0.007**
cg19577958	SERPINB9	3′ UTR	Shelf	0.35 ± 0.04	0.56 ± 0.06	**0.011**	−2.01	0.080
cg07791065	-	Intergenic	—	0.41 ± 0.05	0.62 ± 0.06	**0.006**	−1.73	0.108
cg18232235	RP11-434C1	Promoter	—	0.77 ± 0.04	0.56 ± 0.07	**0.009**	2.39	**0.026**
cg04402345	MFAP3	5′ UTR	—	0.23 ± 0.04	0.44 ± 0.06	**0.013**	−2.82	**0.015**
cg19288863	EYA4	5′ UTR	—	0.56 ± 0.04	0.77 ± 0.04	**0.004**	−3.48	**0.004**

Bold *p*-values indicate statistical significance at α = 0.05. ^a^ Mann-Whitney U test (non-parametric) comparison.

Hannum and Levine epigenetic clocks did not indicate any significant age acceleration or deceleration between the SE groups. Interestingly, however, Epi-Age calculated using the Horvath clock indicated deceleration in individuals with LSE (*p* = 0.042) and age acceleration in individuals with NSE (Table 5).

**TABLE 5:** Mean epigenetic age (Epi-Age) acceleration per SE group. Epi-Age values are represented here as the difference between mean Epi-Age and mean chronological age. Positive scores suggest age acceleration, while negative scores suggest age deceleration.

	Total cohort	Self-esteem (SE) groups		
		NSE	LSE	*p* ^a^
Epi-Age scores (years; mean ± SEM)				
Hannum	−0.50 ± 1.00	0.05 ± 1.38	−1.59 ± 1.24	0.208
Horvath	1.17 ± 1.18	1.95 ± 1.65	−0.36 ± 1.32	**0.042**
Levine (PhenoAge)	1.58 ± 1.41	1.24 ± 2.02	2.26 ± 1.40	0.565

Bold *p*-values indicate statistical significance at α = 0.05.

^a^ Kruskal-Wallis non-parametric ANOVA.

**FIGURE 6:**
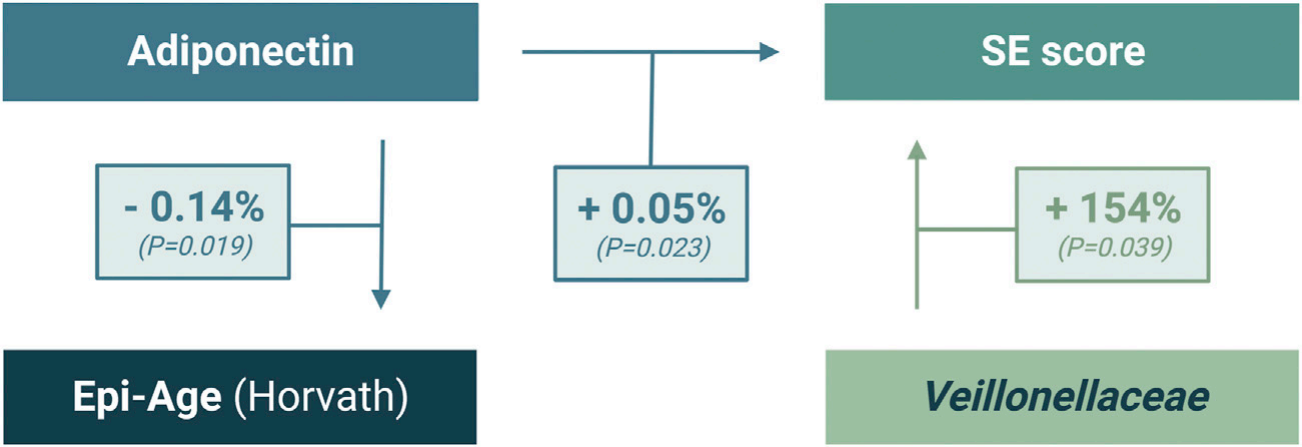
Multiple-regression analysis of significant interaction between SE scores, adiponectin, and gut microbial abundance of *Veillonellaceae.*

Our mediation analysis of multiple immunometabolic and inflammatory biomarkers, microbiota, and Epi-Age (Figure 6) revealed that a 1% difference in plasma levels of adiponectin between individuals associates with significant independent differences in both Epi-Age (Horvath) and SE score by −0.14% (*p* = 0.019) and 0.05% (*p* = 0.023), respectively. While, conditionally, if *Veillonellaceae* is detected (relative abundance is more than zero), a difference in 1% of its relative abundance associates with 1.54-fold difference in the SE score (*p* = 0.039). However, the relative abundance of this taxon was not significantly associated with Epi-Age (Horvath). Additional results of exploratory multivariate regression analyses are provided in Supplementary Table S4.

## Discussion

Self-esteem (SE) has been shown to be an early indicator of other depressive disorders (Steiger et al., 2014). Thus, investigating the pathophysiologic differences associated with low SE (LSE) may aid in the identification of novel biomarkers predictive of depression to enable preventative interventions among high-risk populations that include NHPI. While existing studies have investigated diagnostic biomarkers related to depression itself, no such research has elucidated physiological bases for SE as one of its predictors. This gap in existing data hindered our ability to compare our observations with those from other ethnic groups. Thus, we focused on characterizing differences in the immunoepigenetic-gut microbiome axis in relation to SE from our NHPI-enriched cohort.

Briefly, we observed individuals with LSE to have relatively higher levels of inflammatory biomarkers (Table 2). Furthermore, we observed an inverse relationship between SE and plasma bioavailability of pro-inflammatory factors (i.e., IL-8, IL-6, and TNF-α). Conversely, adiponectin levels exhibited a direct relationship with SE score, which is consistent with existing literature among depressed individuals (Hu et al., 2015). Adiponectin has been previously described to exhibit an inhibitory effect on TNF-α-induced IL-8 synthesis (Kobashi, et al., 2005). Furthermore, adiponectin is thought to increase insulin sensitivity, which is inversely proportional to the amount of insulin the pancreas needs to produce to induce glucose uptake. Our observations suggest that LSE may exacerbate predisposition to immunometabolic dysregulation *via* the inhibitory effects of adiponectin.

We also identified trends in the gut metagenome that may characterize features of depressive symptoms that associate with LSE. Existing literature has described an altered gut microbial composition in depressed individuals (Jiang et al., 2015), however, no studies have investigated such differences in the context of SE in NHPI populations. Exhibiting significance in both intergroup comparison and correlational analysis, the bacteria we have identified in this study (Table 3) may serve as candidate biomarkers of LSE and/or modifiers of SE and warrants further investigation.

In addition to gut bacterial associations with SE, robust differences in monocyte DNA methylation states at specific loci involved in metabolic activity were observed to relate to SE (Table 4). Indeed, we have identified strong associations with monocyte DNA methylation levels at specific loci and neurocognitive impairment (Corley et al., 2016). While the molecular consequences of the differences in methylation at these loci are unclear, monocytes traffic into the central nervous system, differentiate into microglia and induce neuroinflammation. The differentially methylated CpGs observed in our study that associate with LSE could potentially serve as biomarkers of exposures underlying the condition and/or play functional roles in immunometabolic dysregulation observed in LSE individuals. Our gene-ontology (GO) pathway analysis revealed that biological processes related to metabolism were the top hits across the 104 DML associated with SE (Figure 5), which may implicate functional differences in immunometabolic activity in individuals with LSE.

Beyond those directly associated with individual SE scores, DML conventionally representative of Epi-Age (Horvath) exhibited differential methylation upon SE stratification (Table 5). This result may suggest that NHPI individuals with NSE are aging *faster* than those with LSE. Due to the exploratory nature of our initial investigations within this cohort, our analyses are limited by sample size and the cross-sectional nature of our study design. While these limitations restrict the generalizability of this result, the differences we observed with Epi-Age between SE groups have not been previously explored in the context of mental health disparities. Among the NHPI population, age has served as a crucial risk factor for cardiometabolic pathophysiology, resulting in recommendations to stratify NHPI populations by age (Uchima et al., 2019). While the small sample size of our initial investigation limited our capacity to impose such stratification, the differences in Epi-Age between NSE and LSE groups in our cohort warrant further investigation in a larger study with increased representation of the NHPI population and raises additional questions relevant to racial-ethnic health disparities. As an example, the prognostic capacity of quantifiable “biological age” metrics in racial-ethnic groups that are commonly underrepresented in biomedical research could be explored to explain the disproportionately higher prevalence of age-related chronic diseases in such populations.

**FIGURE 7:**
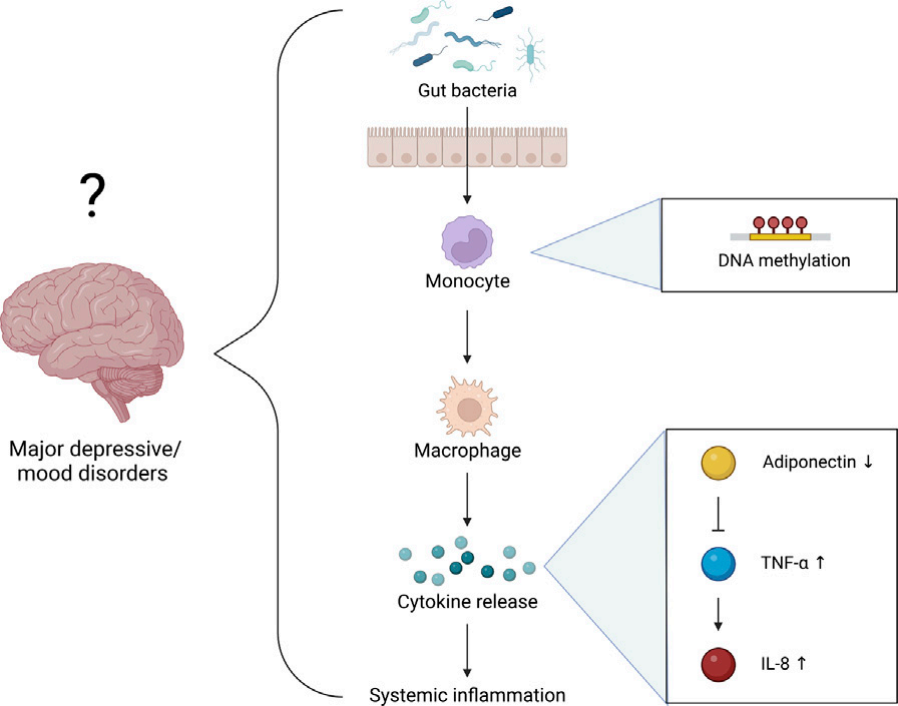
Hypothetical mechanisms underlying the function of SE as an early indicator for the progression of major depressive/mood disorders. We propose that LSE is associated with an epigenetic “priming” of (or increased propensity for) systemic inflammation and/or immunometabolic dysregulation *via* pathways resembling T2DM pathophysiology.

While the functional consequences of the interactions between Epi-Age, SE, and systemic inflammation remain unclear, our regression analyses revealed the possibility of multifaceted, integrated, and/or indirect interactions that characterize individuals with LSE. The relatively miniscule difference (1%) in adiponectin between individuals in our cohort was associated with a statistically significant impact on both the Horvath-derived Epi-Age and SE score (Figure 6). This illustrates a strong interrelationship within the immunoepigentic gut-microbiome brain axis that warrants further examination. Our findings lend support for a hypothesis that immunoepigenetic priming of systemic inflammation as a partial determinant of disparate mental health outcomes in NHPIs, potentially *via* adiponectin-mediated signaling pathways and specific gut microbial interactions (Figure 7). We observed that individuals with LSE showed deficiencies in regulatory activity of adiponectin and subsequent downstream targets involved in inflammation, gut dysbiosis, and differential DNA methylation at immunometabolic genes in monocytes.

Several limitations exist for this study including the small sample size (*N* = 68) of our cohort. Continued efforts to expand this cohort are ongoing. A notable limitation is that the SE score stratification of our cohort is a threshold, as opposed to a continuous variable. SE, as well as other health conditions, are often characterized by a spectrum, not as a categorical measure with a threshold score. This self-reported questionnaire is also subjective, which may contain several potential sources of bias, suggesting that it may not be the most accurate representation of one’s mental state. Additionally, our cohort did not have a medical diagnosis of depression or other mental illness. However, using SE as a proxy variable for mental wellbeing was implemented to account for cultural stigmatization surrounding mental illness and unequal accessibility to professional healthcare to obtain such diagnoses. Thus, we cannot assume that there is a direct correlation between LSE and depressive disorders in NHPIs. However, SE has been previously described to be strongly correlated with depression in a similar NHPI-enriched cohort of approximately 2,000 participants (Juarez et al., 2022). Another limitation of this study is the application of the Infinium MethylationEPIC BeadChip (Illumina) used for the DNA methylation analysis, as is limited to surveying less than 3% of the total CpG sites within the human genome. Future studies could employ techniques that offer a more extensive coverage of CpGs, such as whole genome bisulfite sequencing.

In summary, we have identified the molecular characteristics underlying LSE in a cohort enriched with NHPI. These associative data suggest a role for the immunoepigenetic-gut microbiome axis in SE. The modifiable component of epigenetic processes and the gut microbiome makes this axis an attractive target for future potential therapeutics. As LSE is an early indicator of major depressive/mood disorders, preventive measures could be improved by targeting this axis given a better understanding of causal relationships. However, this study investigated the correlations between SE and physiological biomarkers, which neither imply causation nor explain the high suicide mortality rate or prevalence of depressive symptoms in NHPI communities. Our initial findings warrant further investigation to better understand the causal relationships between inflammation, gut microbial composition, and epigenetics in the context of SE and other mental health conditions in a longitudinal study.

## Data Availability

All data used for this project will be available de-identified when approved by the University of Hawaii Institutional Review Board upon reasonable request to the corresponding author. The gut microbiome data presented in the study are deposited in the figshare repository, accessible at: https://doi.org/10.6084/m9.figshare.21809838.v1. The monocyte DNA methylation data are deposited on the Gene Expression Omnibus (GEO-NCBI) under access number GSE222131.
