# Supplementary figures and images for: Examining the immunoepigenetic-gut microbiome axis in the context of self-esteem among Native Hawaiians and other Pacific Islanders
Source: Front Genet. 2023 Apr 19;14:1125217. doi: 10.3389/fgene.2023.1125217 (PMC10154580; doi:10.3389/fgene.2023.1125217)

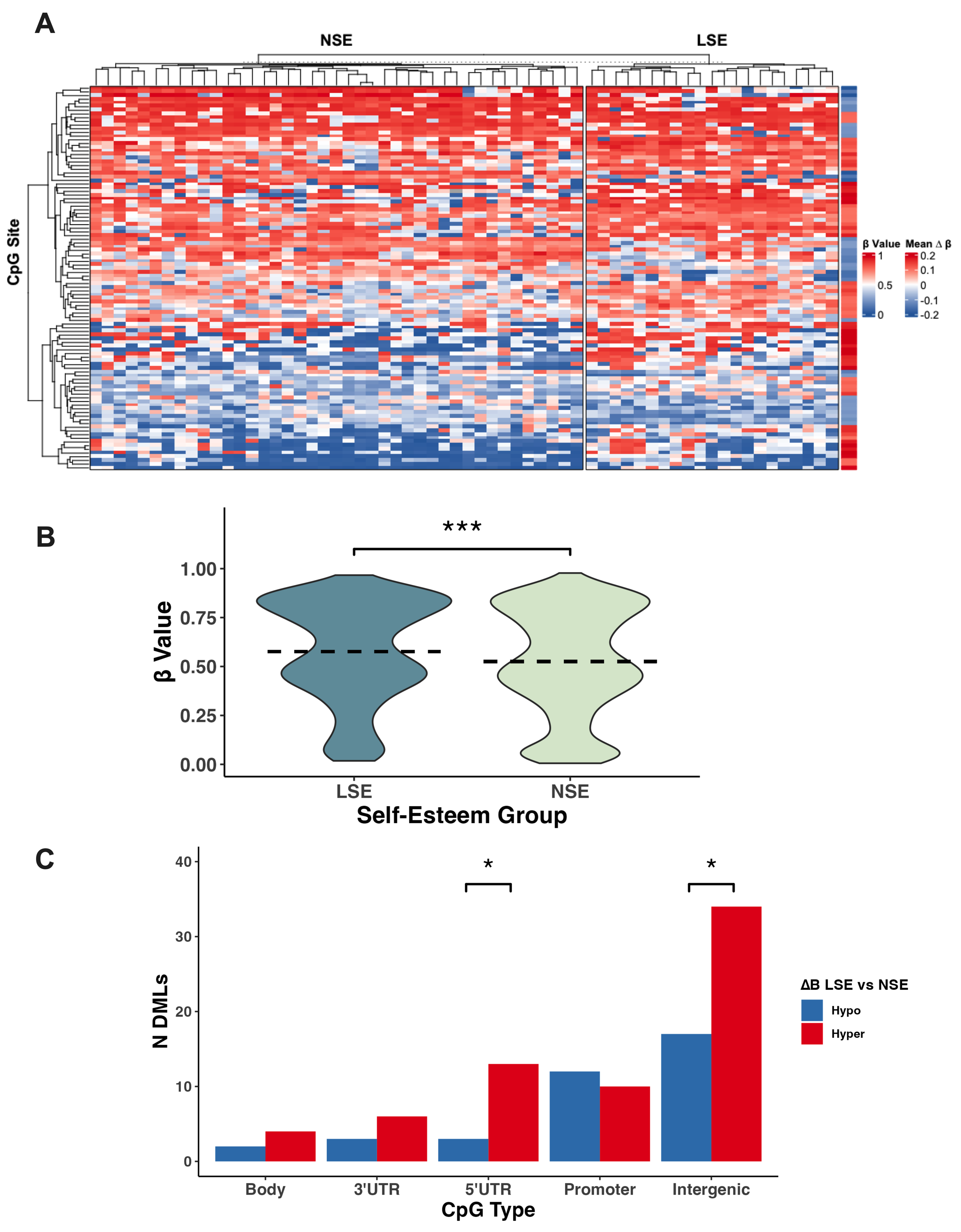

Supplement: Supplementary file 1 [file Image1.TIFF]

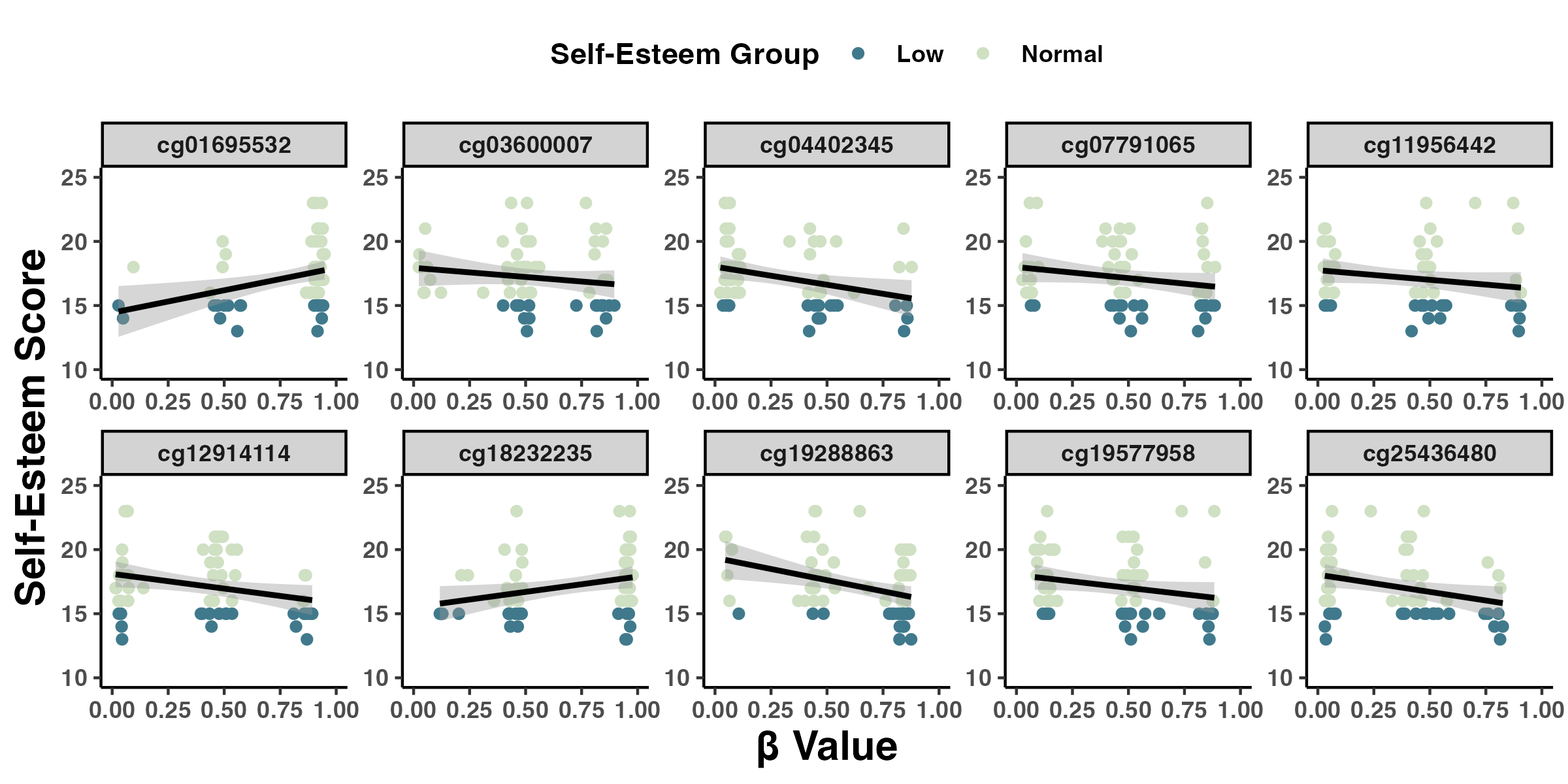

Supplement: Supplementary file 3 [file Image2.TIFF]
