# Supplementary material for: Examining the immunoepigenetic-gut microbiome axis in the context of self-esteem among Native Hawaiians and other Pacific Islanders
Source: Front Genet. 2023 Apr 19;14:1125217. doi: 10.3389/fgene.2023.1125217 (PMC10154580; doi:10.3389/fgene.2023.1125217)
Supplement: Supplementary file 2 [file Table1.DOCX]

**Table S1.** Sample questions utilized for the collection of Rosenberg Self-Esteem data. Likert-scale point-value attributions are indicated below each possible response. A collective score of ≤15 indicates LSE, and that of >15 indicates NSE.

| **Positive attitudes** | **Strongly Disagree** | **Disagree** | **Agree** | **Strongly Agree** |
| --- | --- | --- | --- | --- |
| I feel that I'm a person of worth, at least equal to others. | 0 | 1 | 2 | 3 |
| I feel that I have a number of good qualities. | 0 | 1 | 2 | 3 |
| I take a positive attitude toward myself. | 0 | 1 | 2 | 3 |
| I am able to do things as well as most other people. | 0 | 1 | 2 | 3 |
| On the whole, I am satisfied with myself. | 0 | 1 | 2 | 3 |
|  |  |  |  |  |
| **Negative attitudes** | **Strongly Disagree** | **Disagree** | **Agree** | **Strongly Agree** |
| All in all, I am inclined to feel that I am a failure. | 3 | 2 | 1 | 0 |
| I feel I do not have much to be proud of. | 3 | 2 | 1 | 0 |
| I wish I could have more respect for myself. | 3 | 2 | 1 | 0 |
| I certainly feel useless at times. | 3 | 2 | 1 | 0 |
| At times I think I am not good at all. | 3 | 2 | 1 | 0 |

**Table S2.** Intergroup comparisons and linear regression analyses for gut bacterial α-diversity with respect to SE.

|  | Self-Esteem (SE) Groups | | | | | | |  |  | SE Score Multi-Regression | |
| --- | --- | --- | --- | --- | --- | --- | --- | --- | --- | --- | --- |
|  | NSE | | | LSE | | | P^A^ |  |  | R^2^ | P |
| **Family-level α-diversity** (mean ± SEM) | | | | | | | |  |  |  |  |
| Chao-1 | 25.16 | ± | 1.03E+00 | 26.91 | ± | 1.63E+00 | 0.466 |  |  | -0.09 | 0.457 |
| Shannon | 2.26 | ± | 7.51E-02 | 2.29 | ± | 1.14E-01 | 0.689 |  |  | -0.09 | 0.471 |
| Simpson | 0.80 | ± | 1.28E-02 | 0.81 | ± | 1.54E-02 | 0.680 |  |  | -0.10 | 0.402 |
| **Genus-level α-diversity** (mean ± SEM) | | | | | | | |  |  |  |  |
| Chao-1 | 30.84 | ± | 1.09E+00 | 31.52 | ± | 1.19E+00 | 0.491 |  |  | -0.06 | 0.627 |
| Shannon | 2.22 | ± | 7.40E-02 | 2.27 | ± | 1.11E-01 | 0.748 |  |  | -0.05 | 0.706 |
| Simpson | 0.77 | ± | 1.47E-02 | 0.78 | ± | 1.68E-02 | 0.728 |  |  | -0.09 | 0.493 |
| **Species-level α-diversity** (mean ± SEM) | | | | | | | |  |  |  |  |
| Chao-1 | 51.96 | ± | 2.30E+00 | 54.44 | ± | 3.08E+00 | 0.309 |  |  | -0.17 | 0.160 |
| Shannon | 2.63 | ± | 1.02E-01 | 2.75 | ± | 1.52E-01 | 0.483 |  |  | -0.11 | 0.393 |
| Simpson | 0.82 | ± | 1.63E-02 | 0.84 | ± | 1.84E-02 | 0.808 |  |  | -0.07 | 0.582 |

Bold P-values indicate statistical significance at α=0.05. Italicized P-values indicate concurrent significance among intergroup comparisons and correlation analyses. ^A^Mann-Whitney U test (unpaired, nonparametric, two-tailed).

**Table S3.** 16s metagenomic sequencing quality control table (N=67).

| **Total Reads (10^3^)** | **Mean Bases (Mb)** | **Mean Read Length (bp)** | **Mean Q20 Bases (%)** |
| --- | --- | --- | --- |
| 172730 ± 16850 | 37 ± 3.80 | 216 ± 1.11 | 88 ± 0.001 |

**Table S4.** Multiple linear regression results.

|  | **SE score** | **Epi Age (Horvath)** |
| --- | --- | --- |
| **Immunometabolic hormone** (R ± SE) | | |
| TNF-α | -0.015 ± 0.022 | -0.074 ± 0.057 |
| Adiponectin | 0.053 ± 0.023 (*) | -0.139 ± 0.056 (*) |
| **Relative abundance of identified taxa** (R ± SE) | | |
| *Veillonellaceae* | 1.540 ± 0.72 (**) | -1.449 ± 1.85 |
| *Clostridiales.Family.XIII..Incertae.Sedis* | 3.301 ± 99.3 | -41.84 ± 260.3 |
| *Syntrophomonadaceae* | 186.2 ± 278.4 | -1,477.7 ± 1106.4 |
| *Oxalobacteraceae* | -0.228 ± 0.854 | 2.533 ± 2.123 |
| *Verrucomicrobiaceae* | 9.242 ± 47.97 | 190.502 ± 118.8 |
| Constant | 2.818 ± 0.071 (**) | 3.545 ± 0.18 (**) |
| Observations | 46 | 42 |
| R^2^ | 0.475 | 0.454 |
| Adjusted R^2^ | 0.325 | 0.278 |
| Residual Std. Error | 0.117 (df = 35) | 0.289 (df = 31) |
| F Statistic | 3.169** (df = 10; 35) | 2.580* (df = 10; 31) |
| Levels of statistical significance are denoted as follows: * (*P<0.05*); ** *(P<0.01*). | | |
